# Design of a Radial Vortex-Based Spin-Torque Nano-Oscillator in a Strain-Mediated Multiferroic Nanostructure for BFSK/BASK Applications

**DOI:** 10.3390/mi13071056

**Published:** 2022-06-30

**Authors:** Huimin Hu, Guoliang Yu, Yiting Li, Yang Qiu, Haibin Zhu, Mingmin Zhu, Haomiao Zhou

**Affiliations:** 1 Key Laboratory of Electromagnetic Wave Information Technology and Metrology of Zhejiang Province, College of Information Engineering, China Jiliang University, Hangzhou 310018, China; hm_hu21@163.com (H.H.); glyu@cjlu.edu.cn (G.Y.); li_yiting_lyt@163.com (Y.L.); qiuyang@cjlu.edu.cn (Y.Q.); 2 Jiaxing Key Laboratory of Flexible Electronics Based Intelligent Sensing and Advanced Manufacturing Technology, Institute of Flexible Electronics Technology of THU, Jiaxing 314006, China; zhuhaibin@ifet-tsinghua.org

**Keywords:** spin torque nano-oscillations, radial vortex, multiferroic, BFSK, BASK

## Abstract

Radial vortex-based spin torque nano-oscillators (RV-STNOs) have attracted extensive attention as potential nano microwave signal generators due to their advantages over other topological states, such as their higher oscillation, higher microwave power, and lower power consumption. However, the current driving the oscillation frequency of the STNOs must be limited in a small range of adjustment, which means less data transmission channels. In this paper, a new RV-STNO system is proposed with a multiferroic nanostructure, which consists of an ultrathin magnetic multilayer and a piezoelectric layer. Phase diagrams of oscillation frequency and amplitude with respect to piezostrain and current are obtained through micromagnetic simulation. The results show that the threshold current density of −4000-ppm compressive strain-assisted RV-STNOs is reduced from 2 × 10^9^ A/m^2^ to 2 × 10^8^ A/m^2^, showing one order of magnitude lower than that of conventional current-driven nano-oscillators. Meanwhile, the range of oscillation frequency adjustment is significantly enhanced, and there is an increased amplitude at the low oscillation point. Moreover, a promising digital binary frequency-shift key (BFSK) and binary amplitude-shift key (BASK) modulation technique is proposed under the combined action of current pulse and piezostrain pulse. They can transmit bit signals and show good modulation characteristics with a minimal transient state. These results provide a reference for developing the next generation of spintronic nano-oscillators with a wide frequency range and low power consumption, showing potential for future wireless communication applications.

## 1. Introduction

Much effort has been devoted to the study of spin-torque nano-oscillators (STNOs), which have potential applications in spintronic-based microwave signal generators and microwave detectors [1,2,3,4]. In STNOs, the orientations of magnetization are excited into a steady state of periodic oscillation by the spin-transfer torque effect [5,6,7]. The oscillatory magnetization can be translated into a time-varying *t* output-electrical signal via the magnetoresistance effect [8,9]. However, STNOs based on the uniform precession of magnetization suffer the disadvantages of low output power and large emission linewidth. Various solutions to these problems have been proposed. One effective way to reduce the spectral linewidth is to use the topological soliton oscillation induced by a spin-polarized current as the source of microwave power, such as magnetic droplet [10,11], circular vortex [12,13], skyrmion [14,15], and domain wall [16,17]. These STNOs are based on the circular motion of the magnetic vortex and have the advantages of small size, wide operating frequency, low power consumption, high sensitivity, and easy integration, thereby overcoming the shortcomings of precession-based STNOs [18]. Among the magnetic solitons, circular vortex-based STNOs (CV-STNOs) exhibit excellent performance, such as narrow linewidths (~0.3 MHz) and high quality factors (~4000) [19], as well as emission power of up to ~10 μW [20]. A novel topological structure with radial chirality that has recently attracted much attention is the radial vortex [21,22], which is stabilized by introducing an interfacial Dzyaloshinskii-Moriya interaction (i-DMI). The magnetization of the radial vortex is composed of an out-of-plane component that represents the core polarity and in-plane components for radial chirality, where the chirality outside the core region is fixed to the core polarity. This means that as the core polarity is reversed to the opposite direction, the full in-plane-magnetization components will follow the variation in the radial vortex structure [23,24]. Therefore, compared with CV-STNOs, the radial vortex-based STNOs (RV-STNOs) can provide a greater microwave power emission at a lower current density, since the radial vortex core (RVC) shows a larger amplitude of magnetization oscillations when it has the same orbit radius of periodic gyrotropic motion as the circular vortex core [23]. Meanwhile, compared with a circular vortex, it needs a lower threshold current density (<10^10^ A/m^2^) to expel the RVC from the central region of the nanodisk to follow a periodic gyrotropic motion [21].

Another approach to decreasing the output linewidth is to improve the phase noise characteristics with a phase locking design [25,26,27]. STNO-modulation schemes utilizing frequency-shift keying [25,28] or amplitude-shift keying [3] have been presented to circumvent the issue of large linewidth. The digital signal used in the modulation does not require a radio-frequency mixer in demodulation, and the data can be encoded by two distinct frequencies or two distinct oscillation amplitudes, approaches that are defined as binary frequency-shift keying (BFSK) or binary amplitude-shift keying (BASK), respectively. The FSK and ASK modulations for STNOs based on magnetization precession have been intensively demonstrated by controlling an injected DC current, since it allows for particularly simple and compact device designs that can be easily integrated [28,29]. In these works, the actual modulation method is on-off keying and the modulation speed can be as high as 1.48 Gbps with a theoretical 1 ns turn-on time [3]. In STNOs-based on a vortex magnetic tunnel junction, the modulation frequency of FSK can be up to 10 MHz with data rates of 20 Mbps [30]. The combined effect of the wide range of frequency tenability and the nonlinearity-driven amplitude-frequency modulation limits the application of STNOs in nano-sized microwave frequency modulators [31]. Only using current driving in an STNO cannot fully meet the demands for wide frequency-modulation bandwidth, ultrafast operation, and high data rate in wireless communication applications. The power consumed by using a modulated current or magnetic bias field in a nano-sized STNO is also a challenge for future compact spintronic devices.

The strain-mediated magnetoelectric coupling mechanism in multiferroic hybrids extends the opportunity for manipulation of spin dynamics in spintronic devices, which enables full voltage control of the magnetization orientation at low energy consumption levels [15,32]. In this work, an RV-STNO based on a multiferroic nanostructure is proposed, which excites a circular motion of the RVC by cooperation of both current pulses and piezostrain (voltage) pulses. Micromagnetic simulations show that with the assistance of piezostrain in a current-driven RV-STNO, the oscillation frequency range can be extended and oscillation amplitude improved. The threshold current density of RV-STNOs can be reduced by one order of magnitude by the co-action of compressive piezostrain and current. Phase diagrams of tunable motion frequency and precession amplitude as a function of current and piezostrain are well established for RV-STNOs. In addition, ideal BFSK and BASK behaviors have been digitized using the combined action of current pulse and strain pulse in the control of oscillation frequency and amplitude, respectively. In the present study, we computationally demonstrate a promising method for achieving current-induced oscillation of an RVC as a source of microwave emission with the help of voltage-induced strain, which shows good modulation of BFSK and BASK features without any transient state.

## 2. Model and Simulation

**Figure 1:**
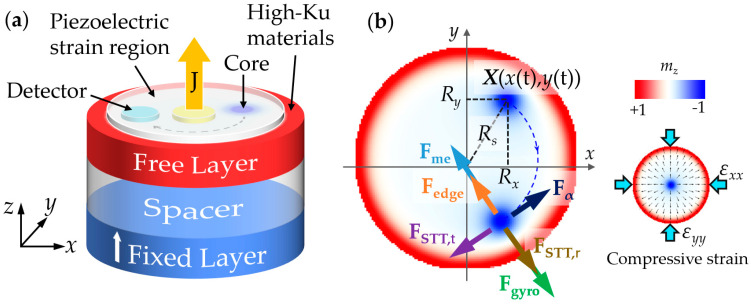
(**a**) Structural model of the STNO based on radial vortex; (**b**) top-view schematic diagram of an analysis of the different forces on the radial vortex core during gyration.

The model structure considered here is a typical magnetic tunnel junction formation of a fixed layer/non-magnetic spacer/free magnetic layer with two nanoscale point-contact electrodes and an additional piezoelectric layer (Figure 1a), in which the fixed layer is perpendicularly magnetized along the +*z*-axis. The free magnetic layer is composed of a bilayered-Co/Pt-based nanodisk surrounded by an annular high-anisotropy material region with a Nd_2_Fe_14_B composition [33]. By offering an upper limit with edge repulsion force, the high-anisotropy ring confines the RVC to the nanodisk and avoids its annihilation at the edge. The i-DMI and the perpendicular magnetic anisotropy formed at the interface between the ferromagnetic Co film and heavy-metal Pt film can generate and stabilize a radial vortex in the nanodisk [34]. The two electrodes are used to inject spin-polarized current with different driving amplitudes and to monitor the magnetization motion of the RVC with a magnetoresistive effect, respectively. With an applied-voltage action, the piezoelectric layer can build a piezostrain region by providing modulated strain pulses. Therefore, this RV-STNO can be well operated by current-driven RVC motion and modulated by the piezostrain-transfer-induced magnetoelastic anisotropy variation of the ferromagnetic layer via inverse magnetostriction [15].

The gyration dynamics of the RVC under the coaction of spin-polarized current and piezostrain can be explored by using the open-source micromagnetic simulation software Mumax^3^. This can be consider the i-DMI, spin-transfer torque, and magnetoelastic contributions [35], in which the Landau-Lifshitz-Gilbert-Slonczewski (LLGS) equation is numerically solved to describe the time-dependent magnetization dynamics [14,36]:

(1) $$\frac{dm}{dt}=-\frac{\gamma }{1+\alpha^{2}}(m\times H_{eff}-\alpha (m\times (m\times H_{eff})))-\tau_{ST}$$

where $m=\left(m_{x},m_{y},m_{z}\right)$ is the magnetic unit vector in the free layer, *γ* is the gyromagnetic ratio, and α is the Gilbert damping constant. The effective field ***H****_eff_* consists of an exchange coupling field, magnetocrystalline anisotropy field, demagnetization field, i-DMI effect field and magnetoelastic field [24]. Slonczewski’s spin-transfer torque term $\tau_{ST}=\frac{\hbar J}{eM_{s}d}g(\theta )\left(\beta (m\times p)-\left(m\times (m\times p)\right)\right)$ is induced by spin current and leads to the generation of RVC gyration motion [37], where *ħ* is the reduced Planck constant, *J* is the injected current density, *e* is the electron charge, *M_s_* is saturation magnetization, *d* is the thickness of the free layer, *g(θ)* is Slonczewski’s expression, *β* is the coefficient of the perpendicular torque (usually taken to be equal to *α*), and ***p*** is the polarization vector of the fixed layer.

To further investigate the effect of strain on the RVC gyration motion, we use the modified Thiele equation (reported in our previous work) to analyze the different forces acting on the RVC, as shown in Figure 1b [15]:

(2) $$G\times \frac{dX}{dt}+\frac{\partial W}{\partial X}+\alpha D\frac{dX}{dt}+F_{STT}+F_{me}=0$$

In the equation, the first term is the gyrovector force ***F****_gyro_*, where $G=-4\pi Qd\mu_{0}M_{s}/\gamma e_{z}$ is the gyromagnetic coupling vector with the topological charge Q=∬q dxdy/4π, q=m⋅[(∂m/∂x)×(∂m/∂y)], where *μ_0_* is the permeability of the free space, and ***e****_z_* is the direction along *z*-axis. the core position is X=X(x(t),y(t)), as shown in Figure 1b. The second term is the repulsive force ***F****_edge_*, where ***W*** is the potential of off-center radial vortex due to the nanodisk-edge effect. The third term represents the damping force ***F****_α_* and *D* is the i-DMI constant. The spin torque force ***F****_STT_* is described by $F_{STT,\;i}=-JP\hbar /2e\iint \left(m\times p\right)\cdot \partial_{i}m\;dxdy,\;i=r,t$, where *p* is the spin polarization, and *i* is the radial or tangential direction of the nanodisk. The applied strain determines the magnetoelastic force with $F_{me,ij}=c_{ijkl}\left(\varepsilon_{kl}-\varepsilon_{kl}^{0}\right)$; here, $c_{ijkl}$ (*i*, *j*, *k*, *l* = 1, 2, 3) is the elastic moduli, $\varepsilon_{kl}$ is the total strain, and $\varepsilon_{kl}^{0}$ is the eigenstrain. For simplicity, we assume that the strain distribution is uniform on the nanodisk and neglect the strain variation near its edge [38,39]. Thus in this work the biaxial in-plane strains ($\varepsilon =\varepsilon_{xx}=\varepsilon_{yy}=-\varepsilon_{zz}$) are applied to the nanodisk. When the RVC rotates stably in a circle with a tangential speed *v_t_*, Equation (2) can be divided into tangential and radial components:

(3a) $$F_{\alpha }e_{t}+F_{STT,\;t}e_{t}=0$$

(3b) $$F_{resu,r}=m\frac{v_{t}^{{}^{2}}}{R_{S}}=4\pi^{2}mf^{2}R_{S}=F_{edge}+\eta F_{me}-F_{gyro}-F_{STT,\;r}$$

where *η* = 1 (−1) represents the compressive(tensile) piezostrain, and *m* is the mass per unit area. In the simulation, the oscillation radius $R_{s}=\sqrt{R_{x}^{2}+R_{y}^{2}}$ is the distance from the core to the center of the disk, where the position of the core is $R_{i}=\iint iq\;dxdy/\iint q\;dxdy,\;i=x,y$ in the Cartesian coordinate system of the disk shown in Figure 1b. By Equation (3a), the compensation of the resultant force in the tangential direction decides the radius of RVC motion and results from the competition between damping torque and tangential spin torque. According to Equation (3b), the resultant force in the radial direction determines the motion speed and oscillation frequency of the RVC gyration motion, which is mainly affected by edge repulsive force, magnetoelastic force, radial spin torque force, and gyrovector force.

Since the RVC rotates stably in a circular trajectory under the co-action of current and piezostrain, the steady-value of *R_s_* is taken as the oscillation amplitude, while the oscillation frequency is obtained by applying the fast Fourier transform (FFT) to the stable oscillation part of *R_x_* using a Lorenz peak-fitting curve. The simulated mesh cell dimension is 2 × 2 × 1 nm^3^. The Co/Pt material parameters used are as follows [24]: exchange stiffness *A_ex_* = 1.5 × 10^−11^ J/m, saturation magnetization *M_s_* = 0.58 × 10^6^ A/m, Gilbert damping *α* = 0.02, i-DMI constant *D* = 1.0 mJ/m^2^ and spin polarization ***p*** = 0.4. The material parameters of Nd_2_Fe_14_B [33] are *M_s_* = 1.28 × 10^6^ A/m, *A_ex_* = 7.7 × 10^−11^ J/m and *K_u_* = 430 kJ/m^3^. The magnetoelastic coupling constant is $B_{1}=-3\lambda_{100}\left(C_{11}-C_{12}\right)/2$, where the elastic constants are *C*_11_ = 304 GPa and *C*_12_ = 150 GPa, and the magnetostriction parameter is *λ_s_* = 70 ppm. The free-layer nanodisk is 240 nm in diameter and surrounds a 5-nm-width high-anisotropy ring, where the thickness of the free layer is *d* = 1 nm. The circular piezostrain region is the same diameter as the free layer and fully covers of it. The diameter of point-contact electrodes is 25 nm.

## 3. Results and Discussion

**Figure 2:**
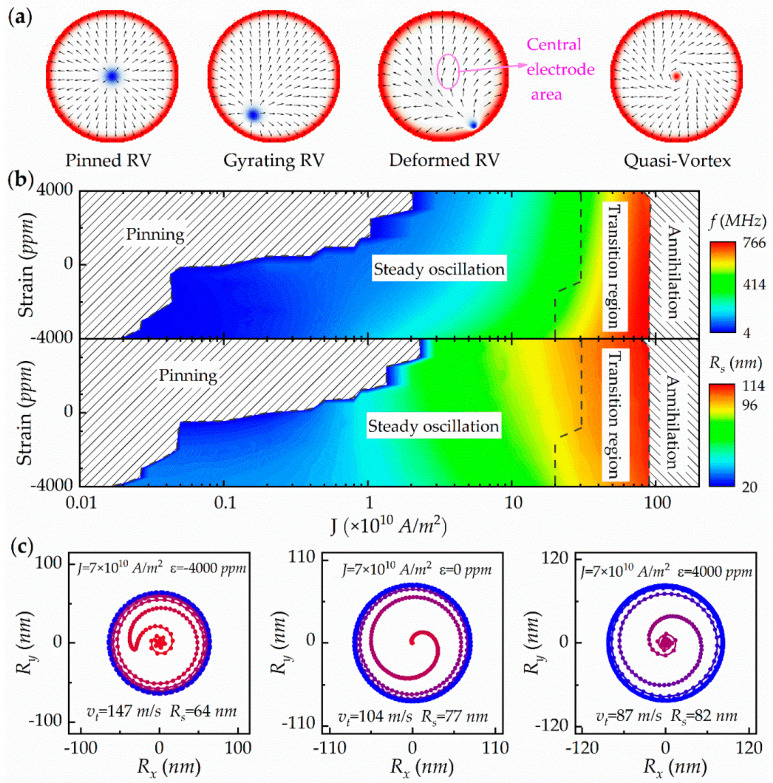
(**a**) Typical topological spin structures in current-driven spin dynamics. (**b**) Phase diagrams of RVC oscillation frequency and oscillation amplitude with respect to current density and piezostrain. (**c**) Trajectory of RVC rotation driven by a fixed current of *J* = 7 × 10^10^ A/m^2^ with assistance by different piezostrains.

Based on our previous work [24], an radial vortex with negative polarity and positive chirality is selected as the initial state, in which the core is at the center of the disk. Figure 2a shows several typical spin structures in the current-driven spin dynamics process. When the current is injected into the center-point-contact electrode, the polarized electrons enter the free layer and transfer the spin-angular momentum to the local magnetization as spin torque. By injecting a small current of *J* < 2 × 10^9^ A/m^2^, the combined force of the polarized current-induced spin torque and gyrovector force in the radial direction is smaller than the repulsive force from the boundary, which leads to the RVC pinning on the center of the disk (first picture in Figure 2a). By increasing the current to exceed the threshold current density of 2 × 10^9^ A/m^2^, the sufficiently large outward radial component induces a clockwise spiral movement of the RVC out of the central region, and finally, it enters into a steady and periodic gyration motion (the second picture in Figure 2a shows the spin structure during the stable rotation). Generally, the polarized current-induced spin torque acts on the local spins from the center electrode to the edge of the disk, so the oversize torque effect may first result in spin deflects of the in-plane magnetized component near the central electrode area when the current exceeds 2 × 10^10^ A/m^2^. This deformed RVC magnetization structure is shown in the third picture in Figure 2a, where it can be seen that the local spins in the magenta circle are obviously deflected. By further increasing the current to *J* > 9 × 10^10^ A/m^2^, the outward current-induced spin torque and gyrovector force will overcome the edge repulsion. This leads to the RVC rotating to the edge of the nanodisk and then being annihilated into a quasi-vortex structure where only the central spins are turned upward by the large current-induced spin torque, while the edge spins stay non-reversed with high-anisotropy-ring pinning, and the middle spins show a clockwise-helical spin distribution (fourth picture in Figure 2a).

Figure 2b shows phase diagrams of the RVC oscillation frequency and oscillation amplitude with respect to current density and piezostrain. Under different combinations of spin current (from 1 × 10^8^ A/m^2^ to 1.5 × 10^12^ A/m^2^) and piezostrain (from −4000 ppm to 4000 ppm), four areas can be divided according to different RVC behaviors (dark grey dotted lines in Figure 2b), namely, pinning, steady oscillation, transition, and annihilation regions. These four areas correspond to the four typical spin structures shown in Figure 2a; however, the piezostrain assist has an influence. In the pinning region, the RVC not only stops at the central point of the disk but may stop at a certain point without oscillation in the disk due to the effect of different piezostrains. In the steady oscillation region, by co-action of spin current and piezostrain, effective ranges of operation frequency and amplitude for the RV-STNO of 4.5–415 MHz and 20–96 nm, respectively, can be achieved. With the help of a −4000-ppm compressive strain, the driving current threshold is reduced from 2 × 10^9^ A/m^2^ to 2 × 10^8^ A/m^2^, while a tensile strain of 4000 ppm increases the threshold current to 3 × 10^10^ A/m^2^. This strain mediated threshold-current variation can be interpreted by Equation (3b). By the application of compressive piezostrain to assist the current driven RVC rotation shown in Figure 2c, the trajectory radius will be reduced but lead to an increase in movement speed, resulting in an enlarged-radial-resultant force. Therefore, among the four-part-component forces in the radial direction, only the combination of increased-compressive strain and decreased-radial spin torque will produce enough radial-resultant force to drive the constrictive RVC rotation with high speed. Conversely, the co-action of tensile strain and current in the RV-STNO will lead to an expanding trajectory with low speed, which requires an increase in tensile strain and also an increase in radial spin torque. Finally, too a high current at all ranges of piezostrain will lead to too large a spin torque, which will induce annihilation of the RVC at the edge, which will enter a quasi-vortex state. There is a transition region between the steady oscillation region and annihilation region, in which the radial vortex state starts to be deformed by the central spins.

**Figure 3:**
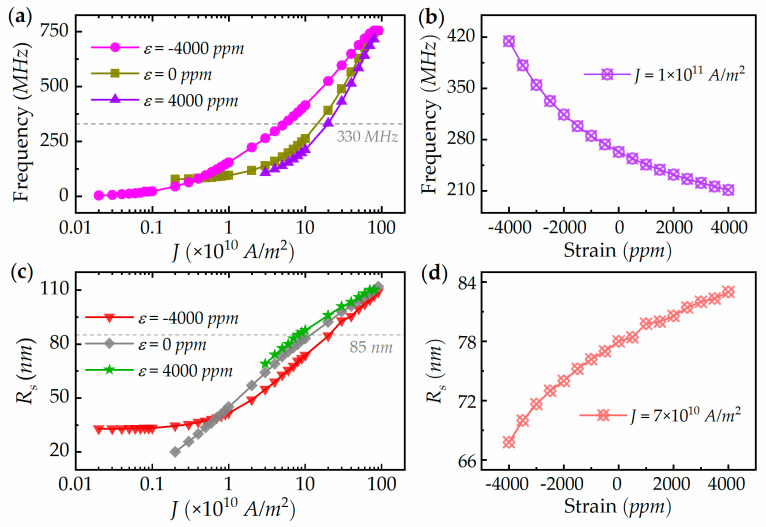
(**a**) Current-dependent oscillation frequency at different piezostrains. (**b**) Piezostrain-dependent oscillation frequency at a fixed current density of *J* = 1 × 10^11^ A/m^2^. (**c**) Current-dependent oscillation amplitude at different piezostrains. (**d**) Piezostrain-dependent oscillation amplitude at a fixed current density of *J* = 7 × 10^10^ A/m^2^.

Figure 3a shows the current-dependent oscillation frequency at three typical piezostrains of −4000 ppm, 0 ppm, and 4000 ppm. In all cases, the RVC gyration frequency increases with current density, which is consistent with that of skyrmion-based STNOs. On one hand, under the compressive piezostrain of −4000 ppm, the range of operation frequency can be increased from 77.3–754 MHz to 4.5–755 MHz compared with the RV-STNO without piezostrain applied. On the other hand, the action of tensile piezostrain has an opposite effect on the frequency adjustment. The intersection of the grey dotted line in Figure 3a represents the current density required under different strains at a given frequency of 330 MHz, which may clearly show the decrease in the driving-current threshold under the assistance of compressive piezostrain, as well as an increase in threshold current by applying a tensile piezostrain. The corresponding piezostrain-dependence of oscillation frequency at a given current density of *J* = 1 × 10^11^ A/m^2^ is shown in Figure 3b. When the piezostrain changes from −4000 ppm to 4000 ppm, the operation frequency can cover 211.3–414.6 MHz. This strain modulated oscillation frequency can be well-expounded with Equation (3b), in which an increase in inward compressive piezostrain will lead to an increase in frequency. The oscillation amplitude is the factor determining the microwave output power, which is represented by the radius of RVC motion. Figure 3c,d show the typical current and piezostrain-dependent RVC-motion radius, respectively. Under the co-action of current density and piezostrain, the radius of RVC motion can be modulated from 20 nm to 114 nm.

**Figure 4:**
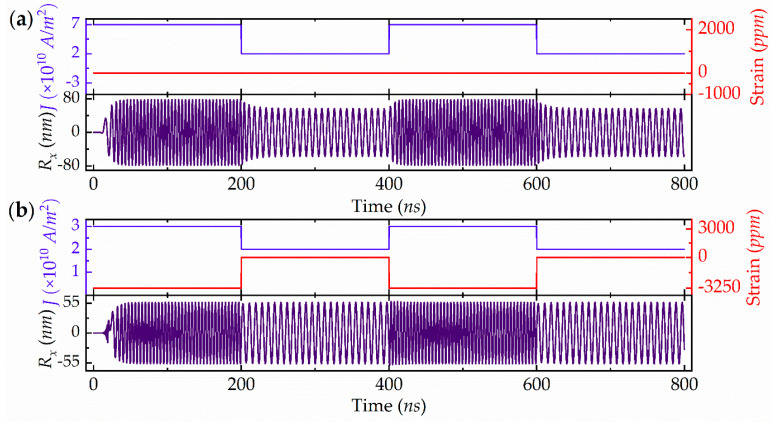
RV-STNO-based BFSK modulation simulations at (**a**) two-distinct current pulses without piezostrain, and (**b**) two distinct current-piezostrain-combined pulses.

The design of ideal BFSK modulation requires switching between two different oscillation frequencies at a fixed amplitude in response to stimuli. Accordingly, a piezostrain and current co-modulated BFSK based on an RV-STNO isproposed. As shown in Figure 4a, by only applying a series of current pulses with alternating amplitudes of 7 × 10^10^ A/m^2^ and 2 × 10^10^ A/m^2^, the frequency can be varied from 221.5 MHz to 118.5 MHz, with the amplitude varying from 78 nm to 57 nm. Generally, the amplitude fluctuation may cause a long transient time between the two modulated frequency states and, thus, limit the data transmission rate [25]. Ideal BFSK modulation needs fast frequency conversion with the transient process absent. Therefore, a cooperative current and piezostrain scheme is chosen to achieve this goal, as shown in Figure 4b. By applying combined current and piezostrain pulses of (3 × 10^10^ A/m^2^, −3250 ppm) and (2 × 10^10^ A/m^2^, 0 ppm), the amplitude frequency can be modulated between 221.5 MHz and 118.5 MHz, which can be used to encode the binary frequency signal. Meanwhile, the amplitude maintains no transition, which strongly suggests that it is a good candidate for BFSK modulation.

**Figure 5:**
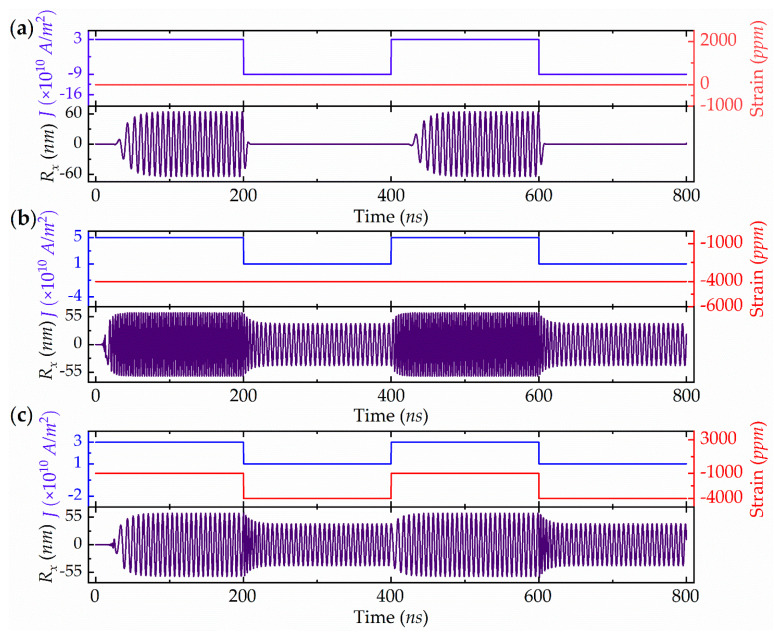
RV-STNO based BASK modulation simulations at (**a**) two-distinct current pulses without piezostrain, (**b**) two distinct current pulses with a fixed piezostrain, and (**c**) two distinct current-piezostrain-combined pulses.

The approach was explored further by designing a BASK modulation system based on a current- and piezostrain-driven RV-STNO. As shown in Figure 5a, by only applying a current pulse of 3 × 10^10^ A/m^2^ to −9 × 10^10^ A/m^2^, a simple BASK behavior can be realized, namely, on-off keying, producing two-amplitude switching from 64 nm to 0 nm at a frequency of 140 MHz. However, this current-driven amplitude switching has a sharp transition over a long time, and a reversed current is needed to shorten the transition time. The current and piezostrain co-modulated BASK behavior are shown in Figure 5b,c. In the first case, a fixed piezostrain of −4000 ppm is applied and the current pulse is varied from 5 × 10^10^ A/m^2^ to 1 × 10^10^ A/m^2^. Under this combination of piezostrain and current pulses, the amplitude can be changed from 62.7 nm to 41.7 nm, but the frequency also shows a variation from 322.2 MHz to 155.2 MHz. This is not ideal BASK modulation behavior. Therefore, in the second case, combined current-piezostrain pulses of (3 × 10^10^ A/m^2^, −1000 ppm) and (1 × 10^10^ A/m^2^, −4000 ppm) are used to modulate the oscillation amplitude. It can be clearly seen in Figure 5c that, by switching the amplitude from 62.7 nm to 41.7 nm, the frequency remains unchanged at a fixed value of 155.2 MHz. Here, the amplitude transition shows a gradual and rapid switching process.

## 4. Conclusions

In conclusion, we propose a radial vortex-based spin-torque nano-oscillator in a strain-mediated multiferroic nanostructure. By applying a proper combination of cooperative current and piezostrain, the stable rotation of the RVC in the nanodisk can be achieved at a low threshold current density of 2 × 10^8^ A/m^2^. The tunable range of motion frequency can be enhanced from 77.3–754 MHz to 4.5–755 MHz by piezostrain assistance in the current-driven RV-STNO. Meanwhile, the oscillation amplitude range can be improved to 33–114 nm, showing a potential improvement in microwave output power. Using established phase diagrams of oscillation frequency and amplitude as functions of current and piezostrain, ideal BFSK and BASK behaviors were designed by the co-action of feasible current and strain pulses. These results may facilitate the design of spintronic nano-oscillators with a novel exotic magnetic structure—radial vortex, and enhance the practical application of STNOs in modern communication systems.

## Data Availability

The data that support the findings of this study are available from the corresponding author upon reasonable request.
